# Severity and Mortality Associated with Steroid Use among Patients with COVID-19: A Systematic Review and Meta-Analysis

**DOI:** 10.1155/2021/6650469

**Published:** 2021-05-06

**Authors:** Tamiru Sahilu, Tadesse Sheleme, Tsegaye Melaku

**Affiliations:** ^1^Department of Pharmacy, College of Health Science, Assosa University, Assosa, Ethiopia; ^2^Department of Pharmacy, College of Health Science, Mettu University, Mettu, Ethiopia; ^3^Department of Clinical Pharmacy, School of Pharmacy, Institute of Health, Jimma University, Jimma, Ethiopia

## Abstract

**Background:**

There are controversial suggestions about steroid use to treat patients infected with COVID-19. Conclusive evidence regarding the use of steroids to treat COVID-19 is still lacking. This meta-analysis aimed to determine the mortality and severity associated with corticosteroid therapy compared to noncorticosteroid treatment in patients with COVID-19.

**Methods:**

The information was collected from electronic databases: PubMed, CINAHL, the Cochrane Library, clinicaltrials.gov, and Google scholar through January 30, 2021. Risk ratios (RRs) with 95% confidence intervals (CIs) were performed using random effect models. Endnote citation manager software version X9 for Windows was utilized to collect and organize search outcomes (into relevant and irrelevant studies) and to remove duplicate articles.

**Results:**

Thirty-two studies were included in the meta-analysis, including 14,659 COVID-19 patients. No significant differences in mortality between the steroid and nonsteroid treatment groups (RR = 0.95; 95% CI: 0.80–1.13; *p* = 0.57). There was no significant reduction in mortality in critically ill COVID-19 patients treated with corticosteroid (RR = 0.89; 95% CI: 0.62–1.27; *p* = 0.52). Significant differences were observed in severe disease conditions between the steroid and nonsteroid treatment groups (RR = 1.10; 95% CI, 1.03–1.19, *p* = 0.007).

**Conclusion:**

There was no significant difference in all-cause mortality between the steroid and nonsteroid treatment users' of COVID-19 patients. There was no significant reduction of all-cause mortality in critically ill COVID-19 patients treated with corticosteroids.

## 1. Background

Coronavirus disease-19 (COVID-19) was first identified at the end of 2019 in Wuhan City, China. It rapidly spread in China and other countries throughout the world [1, 2]. In March 2020, the World Health Organization characterized the disease as pandemic [3]. As of May 21, 2020, more than five million confirmed cases have been documented and several death cases reported globally. It is affecting 213 countries and territories around the world and 2 international conveyances [4]. Severe acute respiratory syndrome coronavirus 2 (SARS-CoV-2) is identified as the cause of COVID-19 [5].

Currently, there is no drug confirmed by clinical trial to prevent or treat COVID-19. However, more than 300 active clinical treatment trials are under investigation [6]. Drugs that are already marketed for other conditions are being off-label used. For example, antimalarial medications chloroquine and hydroxychloroquine are widely used to treat COVID-19 [7]. A multinational registry analysis debunked the benefit of hydroxychloroquine or chloroquine when used alone or with a macrolide. The finding of the study showed that each of these regimens was associated with decreased in-hospital survival and an increased frequency of ventricular arrhythmias when used for the treatment of COVID-19 [8]. Remdesivir is another drug being used as a promising option for treating COVID-19 based on laboratory experiments [9].

Corticosteroids were widely used to treat severe acute respiratory syndrome coronavirus-1(SARS-CoV-1) and Middle East respiratory syndrome coronavirus (MERS) during their outbreaks and are being used in patients with COVID-2019 [10]. Up to 70% of critically ill patients are receiving systemic corticosteroids. It is identified that patients treated with a corticosteroid had more clinical symptoms, a higher inflammation index, and more abnormalities on chest computed tomography [11]. A study showed that the use of high-dose corticosteroids increases the risk of death in patients with severe COVID-19 [12], indicating several controversial issues about the use of the steroid to treat patients infected with COVID-19. It is suggested that available evidence does not endorse the use of a steroid for COVID-19 patients, which may cause several side effects [13]. However, it is believed that short-term glucocorticoid therapy with small- or medium-dose could be beneficial for patients with severe conditions [14]. According to the World Health Organization (WHO) guidelines recommendation, glucocorticoids should only be used under clinical trial conditions [15]. Conclusive evidence regarding the use of the steroid to treat COVID-19 is still lacking. Therefore, this study aims to summarize the current evidence of the severity and mortality associated with steroid therapy for patients with COVID-19 which will support us in making the best decision in the management of the COVID-19.

## 2. Methods

### 2.1. Study Design

Analyses were performed according to the Preferred Reporting Items for Systematic Reviews and Meta-Analyses (PRISMA) guidelines [16]. This study was registered in PROSPERO with registration number CRD42020185773.

### 2.2. Search Strategy

The information was collected from electronic databases: PubMed, CINAHL, the Cochrane Library, clinicaltrial.gov, and Google scholar. There was no limitation applied to the language. The reference list of all identified articles was searched for additional studies. Then, an extensive list of search terms was prepared by the analysis of title, abstract, and keywords of retrieved articles. A full-scale search of databases in PubMed, CINAHL, the Cochrane Central Register of Controlled Trials, clinicaltrial.gov, and Google scholar from inception to January 30, 2021, was performed. No language restriction was imposed during the identification of studies. Flow diagram was used to summarize the number of studies identified, screened, excluded, and finally included in the study.

The search terms used were ((“covid 19” [All Fields] OR “covid 19” [MeSH Terms] OR “covid 19 vaccines” [All Fields] OR “covid 19 vaccines” [MeSH Terms] OR “covid 19 serotherapy” [All Fields] OR “covid 19 serotherapy” [Supplementary Concept] OR “covid 19 nucleic acid testing” [All Fields] OR “covid 19 nucleic acid testing” [MeSH Terms] OR “covid 19 serological testing” [All Fields] OR “covid 19 serological testing” [MeSH Terms] OR “covid 19 testing” [All Fields] OR “covid 19 testing” [MeSH Terms] OR “sars cov 2” [All Fields] OR “sars cov 2” [MeSH Terms] OR “severe acute respiratory syndrome coronavirus 2” [All Fields] OR “ncov” [All Fields] OR “2019 ncov” [All Fields] OR ((“coronavirus” [MeSH Terms] OR “coronavirus” [All Fields] OR “cov” [All Fields]) AND steroidal” [All Fields] OR “steroidals” [All Fields] OR “steroidic” [All Fields] OR “steroids” [MeSH Terms] OR “steroids” [All Fields] OR “steroid” [All Fields]) AND (“mortality” [MeSH Terms] OR “mortality” [All Fields] OR “mortalities” [All Fields] OR “mortality” [MeSH Subheading]) AND (“cohort studies” [MeSH Terms] OR (“cohort” [All Fields] AND “studies” [All Fields]) OR “cohort studies” [All Fields] OR (“cohort” [All Fields] AND “study” [All Fields]) OR “cohort study” [All Fields])) NOT (“review” [Publication Type] OR “review literature as topic” [MeSH Terms] OR “review” [All Fields]).

### 2.3. Study Selection

Two reviewers independently carried out a literature search and examined relevant studies and sequentially screened their titles and abstracts for eligibility. The full texts of potentially eligible studies were retrieved. Disagreements were solved in a discussion. A screening guide was used to ensure that all review authors reliably apply the selection criteria.

### 2.4. Inclusion and Exclusion Criteria

Randomized controlled trials (RCTs), observational studies, prospective and retrospective comparative cohort studies, and case-control studies were eligible for the review. The review considered all articles comparing corticosteroid with noncorticosteroid treatment for patients with the diagnosis of COVID-19. Nonhuman studies and studies that did not report mortality and severity data were excluded from the review. The outcome considered was mortality, death within the hospital (all-cause mortality) of COVID 19 patients. The other outcome studied was the number of severe cases in the two groups (corticosteroid versus noncorticosteroid treatment groups).

### 2.5. Methodological Quality Assessment

Selected papers were assessed by two independent reviewers for methodological validity before inclusion in the review. Observational studies were assessed using Newcastle–Ottawa Scale (NOS), which consists of three domains: (1) subject selection, (2) comparability of the study groups, and (3) assessment of outcome(s). A score of 0–9 was allocated to each study. A standardized Cochrane risk of bias tool was used for the randomized controlled trial (RCT). Any disagreements that arise between the reviewers were resolved through discussion.

### 2.6. Data Extraction

Two reviewers independently extracted data from the studies using a predesigned format prepared. Data that were extracted include first author, a region of study, included population, study design, sample size, comparator group, patient status, age, gender, interventions, mortality, and severity. Severe cases were characterized as patients admitted to ICU or on invasive mechanical ventilation.

### 2.7. Data Analysis and Statistical Methods

To perform a meta-analysis, Review Manager 5.4 (Copenhagen: The Cochrane Collaboration, 2014) was used. The outcome variables were calculated using the Mantel Haenszel formula. Risk ratios (RRs) were reported with 95% confidence intervals (CIs) for the variables. The *p* value was two-tailed, and the statistical significance was set at ≤0.05. Heterogeneity was assessed with the Q-statistic test and the *I*^2^ test. The *I*^2^ statistic measured the percentage of total variation across the studies due to clinical or methodological heterogeneity instead of chance. The significant Q statistics (*p* < 0.05) indicated heterogeneity across the studies; thus, a random effect model was utilized. Substantial heterogeneity was represented by *I*^2^ for >50%.

## 3. Results and Discussion

**Figure 1:**
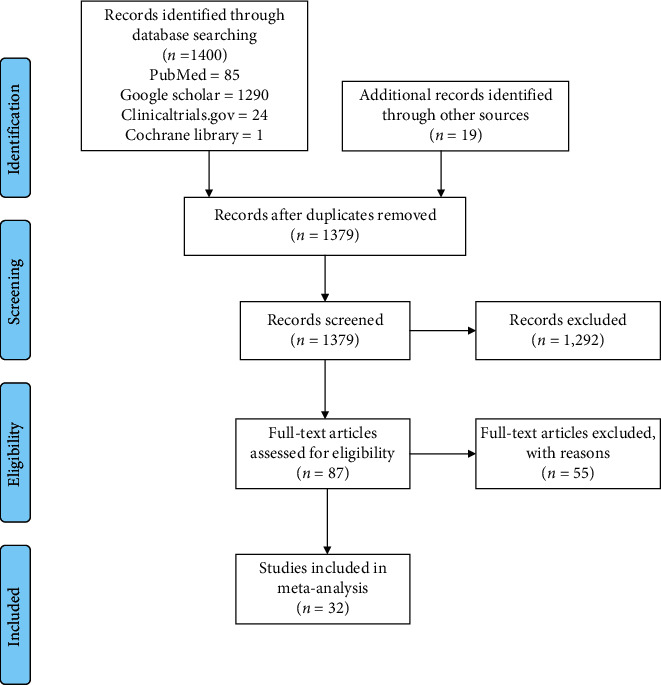
PRISMA study flow diagram.

The search returned a total of 1419 (PubMed: 85, Google Scholar: 1290, clinicaltrials.gov: 24, Cochrane Library: 1, and other sources: 19) citations, of which 40 were duplicates. According to the exclusion criteria, 1292 citations were excluded after the title and abstract screening.  Figure 1 shows the study selection processes.

### 3.1. Characteristics of the Included Studies

The present meta-analysis included 28 observational studies [12, 17–43] and 4 RCTs [44–47]. These studies included 14,659 patients with the diagnosis of COVID-19 who received corticosteroids (5,830 patients) or noncorticosteroids (8,829 patients). Tables 1 and 2 list the characteristics of the included studies. All eligible studies were published in 2020/21. Individual assessment of the risk of bias was presented in Table 3.

### 3.2. Mortality Associated with Steroid Use in Patients with COVID-19

**Figure 2:**
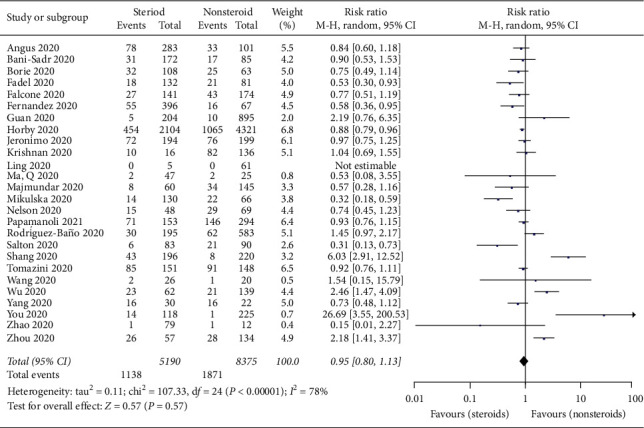
Forest plot for mortality of COVID-19 patients taking steroids versus nonsteroids.

In the 26 included studies [17–19, 21–23, 25–30, 32–35, 37–42, 44, 45, 47, 48] with 13,565 patients, there were no significant differences in mortality between the steroid and nonsteroid treatment groups (RR = 0.95; 95% CI: 0.80–1.13; *p* = 0.57, *I*^2^ = 78%, *p* < 0.0001) (Figure 2). The sensitivity analysis showed that the exclusion of three studies [18, 21, 33] changed the above conclusion.

### 3.3. Mortality Associated with Steroid Use in Patients with Critically Ill COVID-19 Patients

**Figure 3:**
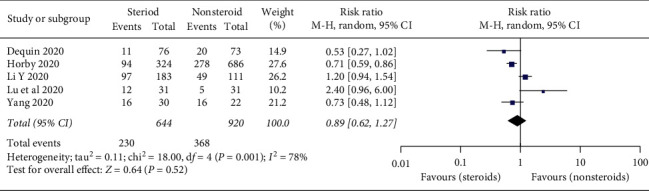
Forest plot for mortality associated with steroid use in patients with critically ill COVID-19 patients.

In the 5 included studies [22, 31, 36, 44, 46] with 1,564 critically ill COVID-19 patients, there were no significant differences in mortality between the steroid and nonsteroid treatment groups (RR = 0.89; 95% CI: 0.62–1.27; *p* = 0.52, *I*^2^ = 78%, *p* = 0.001) (Figure 3).

### 3.4. Severity Associated with Steroid Use in Patients with COVID-19

**Figure 4:**
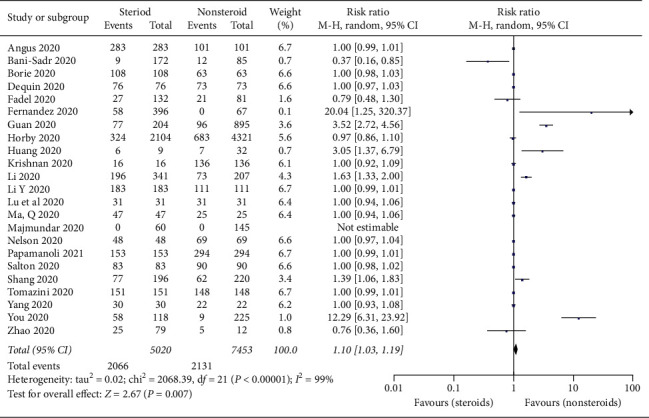
Forest plot for severe events of COVID-19 patients taking steroids versus nonsteroids.

Twenty-three studies reported the severity data of the COVID-19 patients. In the included studies [12, 17, 18, 22, 24, 26–33, 35, 36, 38–40, 42, 44–46, 48] with 12,473 patients, there were significant differences in severe disease condition between the steroid and nonsteroid treatment groups (RR = 1.10; 95% CI, 1.03–1.19, *p* = 0.007) (Figure 4). There was significant heterogeneity among the studies (*I*^2^ = 99% *p* < 0.001); the random-effects model was used.

### 3.5. Publication Bias

**Figure 5:**
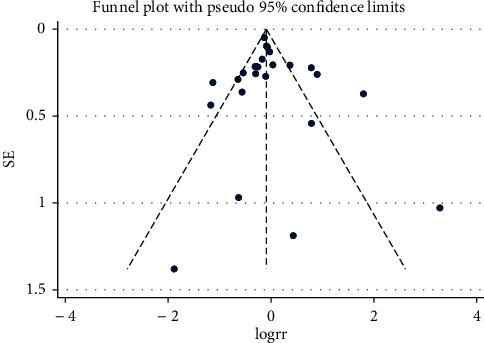
Funnel plot for mortality.

**Figure 6:**
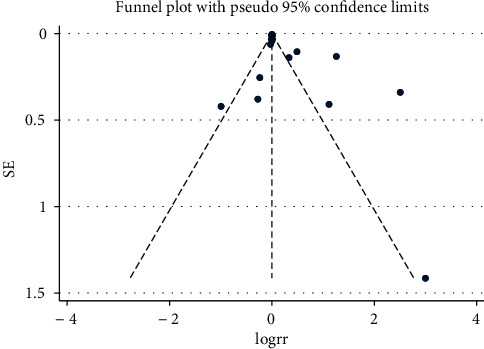
Funnel plot for severe cases.

To assess the small-study effect and publication bias, the regression-based Egger's test was performed. The funnel-plot analysis showed a symmetrical shape for mortality (Figure 5), indicating no publication bias; Egger's test indicated nonsignificant small-study effects (*p* = 0.570). The funnel-plot analysis showed asymmetrical shape for severe cases (Figure 6), indicating publication bias; Egger's test indicated significant small-study effects for severe cases (*p* = 0.027).

### 3.6. Sensitivity Analysis

**Figure 7:**
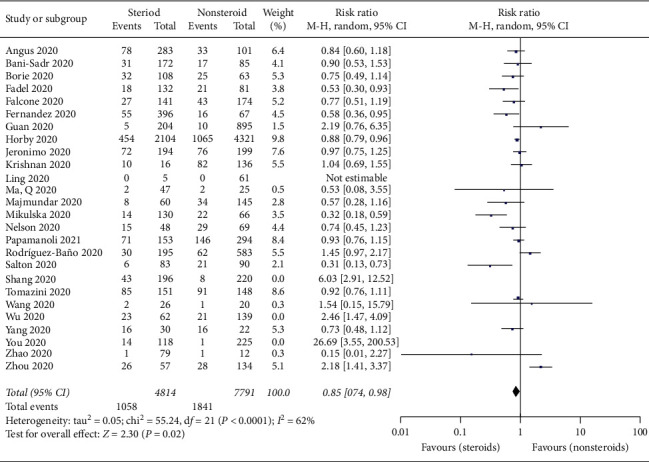
Sensitivity analysis: forest plot for mortality of COVID-19 patients taking steroids versus nonsteroids.

Sensitivity analysis for between-study heterogeneity and analytical methods was performed to show the robustness of the finding. The analysis showed that the result was stable except for the exclusion of three studies [18, 21, 33] that changed the conclusion (Figure 7).

The rationale for the use of corticosteroids is to decrease the host inflammatory responses in the lungs, which may lead to acute lung injury and acute respiratory distress syndrome [6]. Because of the high amount of cytokines induced by COVID-19 infection, corticosteroids were used frequently for the treatment of patients with severe illness, for possible benefit by reducing inflammatory-induced lung injury [24]. There were potential harms and a lack of proven benefit for corticosteroid cautions against their routine use in patients with COVID-19 [6].

Our analysis demonstrated that the mortality rate was comparable in COVID-19 patients treated with corticosteroids versus noncorticosteroids. However, in patients with critically ill COVID-19 patients, even though nonsignificant (RR = 0.89; 95% CI: 0.62–1.27; *p* = 0.52), a lower mortality rate in corticosteroid treatment groups was observed. Evidence suggests that cytokine storm, a hyperinflammatory state resembling secondary hemophagocytic lymphohistiocytosis (HLH), is a contributing factor in COVID-19-associated mortality [49].

In our finding, there were no significant differences in mortality between the corticosteroid and noncorticosteroid treatment groups in critically ill COVID-19 patients. The initial clinical trial results from the United Kingdom (UK) showed that dexamethasone, a corticosteroid, can be lifesaving for patients who are critically ill with COVID-19. For patients on ventilators, the treatment was shown to reduce mortality by about one-third, and for patients requiring only oxygen, mortality was cut by about one-fifth, according to preliminary findings shared with the World Health Organization (WHO) [50].

The most commonly invoked rationale for giving steroids in patients with severe COVID-19 is to modulate the destructive inflammatory immune response that occurs with advancing disease. The guidelines recommend against using corticosteroids to try to modulate the immune system in mechanically ventilated COVID-19 patients without acute respiratory distress syndrome (ARDS) but do recommend steroids in those with COVID-19 and ARDS [51]. Our finding demonstrates that there is an association between severe COVID-19 and corticosteroid therapy (RR = 1.10; 95% CI, 1.03–1.19, *p* = 0.007).

A previous meta-analysis in persons with SARS-CoV-2, SARS-CoV, or MERS-CoV (Middle East respiratory syndrome coronavirus) infection indicated that corticosteroid did not significantly reduce the risk of death [52]. Another meta-analysis that included 5 studies indicated a lack of benefit of corticosteroid therapy on mortality in critically ill patients with COVID-19 [53]. In addition, a review that included 15 studies with coronavirus-infected patients (2 studies with COVID-19 patients) showed that corticosteroid treatment was associated with higher mortality and critical patients were more likely to require corticosteroids therapy [54].

Some of the limitations of this meta-analysis are as follows: the included studies are retrospective cohort studies, there is a high degree of between-study heterogeneity, and mortality may be influenced by other therapeutic options.

## 4. Conclusion

No significant differences in mortality between the corticosteroid and noncorticosteroid treatment groups were observed. There was no significant reduction in mortality in critically ill COVID-19 patients treated with corticosteroids. More randomized clinical trials are needed to further verify this conclusion.

## Figures and Tables

**Table 1:** Characteristics of included studies.

S.No	Authors	Study type	Sample size	Number of control	Patient status	Country	Follow-up period
1	Wang et al. [19]	Retrospective study	46	20	COVID-19 with pneumonia	Wuhan Union Hospital, China	January 20 to February 25, 2020
2	Fadel et al. [17]	Multicenter quasi-experimental study	213	81	COVID-19	Five hospitals in Michigan, USA	March 12, 2020 through March 27, 2020
3	Wu et al. [21]	Retrospective cohort study	201	139	COVID-19 with pneumonia	Wuhan Jinyintan Hospital in China	December 25, 2019, to January 26, 2020
4	Li et al. [12]	Ambispective cohort study	548	207	COVID-19	Tongji Hospital, China	January 26, to March 3, 2020.
5	Zhou et al. [23]	Multicenter, retrospective cohort study	191	134	COVID-19	Wuhan, China	Dec 29, 2019, to Jan 31, 2020
6	Shang et al. [18]	Multicenter, retrospective, observational study	416	220	COVID-19	Hubei province, China	Dec 27, 2019, to Feb 17, 2020
7	Yang et al. [22]	Retrospective observational study	52	22	SARS-CoV-2 pneumonia	Wuhan, China	December, 2019, to Jan 26, 2020
8	Huang et al. [24]	Prospective cohort study	41	32	COVID-19	Wuhan, China	Dec 16, 2019, to Jan 2, 2020
9	Guan et al. [27]	Retrospective cohort study	1099	895	COVID-19	China	December 11, 2019, to January 31, 2020.
10	Zhao et al. [26]	Retrospective cohort study	91	12	COVID-19	Jingzhou Central Hospital, China	January 16, 2020, to February 10, 2020.
11	Ling et al. [25]	Retrospective cohort study	66	61	COVID-19	Shanghai, China	January 20, to February 10, 2020
12	Horby et al. [44]	Randomized controlled trial	6425	4321	COVID-19	United Kingdom	March 9, to June 8, 2020
13	Angus et al. [45]	Randomized controlled trial	384	101	COVID-19	REMAP-CAP multicenter (Australia, Canada, France, Ireland, The Netherlands, New Zealand, the United Kingdom, and the United States)	March 9 to August 12, 2020
14	Borie et al. [28]	Cohort study	171	63	COVID-19	Paris	March 27 to April 10, 2020
15	Dequin et al. [46]	Randomized controlled trial	149	73	Critically Ill patients with COVID-19	France	March 7 to June 29, 2020
16	Falcone et al. [43]	Prospective observational study	315	174	COVID-19 and pneumonia	University Hospital of Pisa	March 4–April 30, 2020
17	Fernández-Cruz et al. [29]	Retrospective controlled cohort study	463	67	COVID-19 and pneumonia	Spain	4 March 2020 to 7 April 2020
18	Jeronimo et al. [47]	Randomized controlled trial	393	199	COVID-19	Brazil	18 April to 16 June 2020
19	Krishnan et al. [30]	Retrospective observational study	152	136	COVID-19 and pneumonia	USA	March 10, to April 15, 2020
20	Li et al. 2020 [31]	Multicenter, retrospective study	294	111	Critically ill COVID-19 patients	Hubei, China	Between December 30, 2019 and February 19, 2020
21	Papamanoli et al. [32]	Retrospective cohort	447	294	severe COVID-19 pneumonia	New York, USA	1 March to 15 April 2020
22	Tomazini et al. [48]	Randomized controlled trial	299	148	Acute respiratory distress syndrome and COVID-19	Brazil	April 17 to July 21, 2020
23	You et al. [33]	Retrospective cohort study	343	225	COVID-19	China	February 1 to March 31, 2020
24	Rodríguez-Baño et al. [34]	Retrospective cohort study	778	583	COVID-19	Spain	February 2 to March 31, 2020
25	Ma et al. [35]	Multicenter retrospective cohort study	72	25	COVID-19	China	January 2020 to March 2020
26	Lu et al. [36]	Retrospective cohort study	62	31	Critically ill COVID-19	China	January 25 to February 25, 2020
27	Cao et al. [37]	Retrospective cohort study	102	51	COVID-19	China	January 3 and February 1, 2020
28	Nelson et al. [38]	Retrospective cohort study	117	69	COVID-19 pneumonia	USA	March 1, 2020 and April 12, 2020
29	Bani-Sadr et al. [39]	Prospective cohort study	257	85	COVID-19 pneumonia	France	3 March 2020 and 14 April 2020
30	Salton et al. [40]	Multicenter observational study	173	90	severe COVID-19 pneumonia	Italy	February 27 to May 21, 2020
31	Mikulska et al. [41]	Observational single-center study	196	66	COVID-19 pneumonia	Italy	NR
32	Majmundar et al. [42]	Retrospective cohort study	205	145	COVID-19 pneumonia	USA	March 15 to April 30, 2020

**Table 2:** Characteristics of included studies.

S. No	Authors	Median age (IQR) in years	Gender	Intervention	No. of patients			
					Mortality		Severe cases	
					Control group	Intervention group	Control group	Intervention group
1	Wang et al. [19]	54 (48–64)	26 (57%) males	Methylprednisolone (*n* = 26)	1	2	NR	NR
2	Fadel et al. [17]	62 (51–62)	109 (51.2%) male	Methylprednisolone (*n* = 132)	21	18	21	27
3	Wu et al. [21]	51 (43–60)	128 (63.7%) men	Methylprednisolone (*n* = 62)	21	23	NR	NR
4	Li et al. [12]	60 (48–69)	279 (50.9%) male	Systemic corticosteroids (*n* = 341)	NR	NR	73	196
5	Zhou et al. [23]	56 (46–67)	Male 119 (62%)	Corticosteroids (*n* = 57)	28	26	NR	NR
6	Shang et al. [18]	49 (36–61)	197 (47%) males	Corticosteroid therapy (*n* = 196)	8	43	62	77
7	Yang et al. [22]	59·7	35 (67%) males	Glucocorticoids (*n* = 30)	16	16	22	30
8	Huang et al. [24]	49 (41–58) years	30 [73%] males	Use of corticosteroid (*n* = 9)	NR	NR	7	6
9	Guan et al. [27]	47 years	639 males	Systemic glucocorticoids(*n* = 204)	10	5	96	77
10	Zhao et al. [26]	46 years	49 males	Glucocorticoid (*n* = 79)	1	1	5	25
11	Ling et al. [25]	44 (34–62) years	38 males	Glucocorticoid (*n* = 5)	0	0	NR	NR
12	Horby et al. [44]	66.1 years	4088 males	Dexamethasone (*n* = 2104)	1065	454	683	324
13	Angus et al. [45]	Mean age, 60 years	29% female	Hydrocortisone (*n* = 283)	33	78	101	283
14	Borie et al. [28]	Median (IQR): 67.1 (56.7–78.1)	Female 48 (28.1%)	Methyl-prednisolone (*n* = 108)	25	32	63	108
15	Dequin et al. [46]	Mean age, 62.2 years	30.2% women	Hydrocortisone (*n* = 76)	20	11	73	76
16	Falcone et al. [43]	Median age was 70 (IQR, 57–80)	(76.2%) males	Steroids (*n* = 141)	43	27	NR	NR
17	Fernández-Cruz et al. [29]	Mean age 66.75 years	317 males	Steroids (*n* = 396)	16	55	0	58
18	Jeronimo et al. [47]	Mean age (SD) 55 ± 15 yrs	139 females	Methylprednisolone (*n* = 194)	76	72	NR	NR
19	Krishnan et al. [30]	68 years (IQR 58–75)	95 males	Oral steroids *n* = 16	82	10	136	16
20	Li et al. [31]	66 yrs (56–75)	197 (67%) males	Corticosteroids, *n* = 183	49	97	111	183
21	Papamanoli et al. [32]	Mean age 61.5 yrs	Females 156	Methylprednisolone, *n* = 153	146	71	294	153
22	Tomazini et al. [48]	Mean age 61.4 yrs	Females 112	Dexamethasone (*n* = 151)	91	85	148	151
23	You et al. [33]	Mean age 53.8	Female 157	Methylprednisolone (*n* = 118)	1	14	9	58
24	Rodríguez-Baño et al. [34]	Age 71 yrs	Female 226	Corticosteroids (*n* = 195)	62	30	NR	NR
25	Ma et al. [35]	Age 60 (13.8) yrs	Female 32 (44%)	Corticosteroid group (*n* = 47)	2	2	25	47
26	Lu et al. [36]	57 (50–69) yrs	Male 32	Steroid (*n* = 31)	5	12	31	31
27	Cao et al. [37]	Age, years 54(37–67)	Female 49	Methylprednisolone Sodium (*n* = 51)	6	11	NR	NR
28	Nelson et al. [38]	Age 61.5 (46–69)	Male 80	Methylprednisolone *n* = 48	29	15	69	48
29	Bani-Sadr et al. [39]	Age 71 yrs	Male 135	Corticosteroids *n* = 172	17	31	12	9
30	Salton et al. [40]	Age 65.75 yrs	Male 120	Methylprednisolone (*n* = 83)	21	6	90	83
31	Mikulska et al. [41]	Age mean 67.5 yrs	Male 132	Methylprednisolone (*n* = 130)	22	14	NR	NR
32	Majmundar et al. [42]	Age, mean 57.61	Male 153	Corticosteroids (*N* = 60)	34	8	0	0

NR: not reported.

**Table 3:** Methodological quality assessment.

Newcastle–Ottawa scale (NOS)									
Studies	Selection				Comparability	Outcome			Quality score
	A	B	c	d	e	f	g	h	
Fadel et al. [17]	*∗*	*∗*	*∗*	*∗*	*∗∗*	*∗*		*∗*	8
Guan et al. [27]	*∗*	*∗*	*∗*	*∗*	*∗∗*	*∗*	*∗*	*∗*	9
Huang et al. [24]	*∗*	*∗*	*∗*	*∗*	*∗*	*∗*	*∗*	*∗*	8
Li et al. [12]	*∗*	*∗*	*∗*		*∗*	*∗*		*∗*	6
Ling et al. [25]	*∗*	*∗*	*∗*	*∗*		*∗*		*∗*	6
Shang et al. [18]		*∗*	*∗*	*∗*	*∗*	*∗*		*∗*	6
Yang et al. [22]	*∗*		*∗*	*∗*	*∗*	*∗*		*∗*	6
Wang et al. [19]	*∗*		*∗*	*∗*	*∗*	*∗*		*∗*	6
Wu et al. [21]	*∗*	*∗*	*∗*	*∗*	*∗*	*∗*		*∗*	7
Zhao et al. [26]	*∗*	*∗*	*∗*	*∗*	*∗*	*∗*			6
Zhou et al. [23]	*∗*	*∗*	*∗*	*∗*	*∗*	*∗*		*∗*	7
Borie et al. [28]	*∗*		*∗*	*∗*	*∗*	*∗*	*∗*		6
Falcone et al. [43]	*∗*	*∗*	*∗*	*∗*	*∗*	*∗*	*∗*		7
Fernández-Cruz et al. [29]	*∗*	*∗*	*∗*	*∗*	*∗*	*∗*	*∗*		7
Krishnan et al. [30]	*∗*	*∗*	*∗*	*∗*	*∗*	*∗*			6
Li et al. [31]	*∗*	*∗*	*∗*	*∗*	*∗*	*∗*			6
Papamanoli et al. [32]	*∗*	*∗*	*∗*	*∗*	*∗*	*∗*	*∗*		7
You et al. [33]	*∗*	*∗*	*∗*	*∗*	*∗*	*∗*			6
Rodríguez-Baño et al. [34]	*∗*	*∗*	*∗*	*∗*	*∗*	*∗*		*∗*	7
Ma, Q et al. [35]	*∗*	*∗*	*∗*	*∗*	*∗*	*∗*	*∗*		7
Lu et al. [36]	*∗*	*∗*	*∗*	*∗*	*∗*	*∗*		*∗*	7
Cao et al. [37]	*∗*	*∗*	*∗*	*∗*	*∗*	*∗*			6
Nelson et al. [38]	*∗*	*∗*	*∗*	*∗*	*∗*	*∗*		*∗*	7
Bani-Sadr et al. [39]	*∗*	*∗*	*∗*	*∗*	*∗*	*∗*			6
Salton et al. [40]	*∗*	*∗*	*∗*	*∗*	*∗*	*∗*		*∗*	7
Mikulska et al. [41]	*∗*	*∗*	*∗*	*∗*	*∗*	*∗*			6
Majmundar et al. [42]	*∗*	*∗*	*∗*	*∗*	*∗*	*∗*		*∗*	7

*Cochrane risk of bias tool*									
	I	J	k		l		m		n
Horby et al. [44]	Low risk of bias	Low risk of bias	High risk of bias		Unclear risk of bias		Low risk of bias		Low risk of bias
Angus et al. [45]	Low risk of bias	Low risk of bias	High risk of bias		Low risk of bias		Low risk of bias		Low risk of bias
Dequin et al. [46]	Low risk of bias	High risk of bias	High risk of bias		Low risk of bias		Low risk of bias		Low risk of bias
Jeronimo et al. [47]	Low risk of bias	Low risk of bias	High risk of bias		Low risk of bias		Low risk of bias		Low risk of bias

a: representativeness of the exposed cohort, b: selection of the nonexposed cohort, c: ascertainment of exposure, d: demonstration that the outcome of interest was not present at the start of the study, e: comparability of cohorts based on the design or analysis, f: assessment of outcome, g: follow-up long enough for outcomes to occur, h: adequacy of follow-up of the cohort; i: random sequence generation (selection bias), j: allocation concealment (selection bias), k: blinding of participants and personnel (performance bias), l: blinding of outcome assessment (detection bias), m: incomplete outcome data (attrition bias), n: selective reporting (reporting bias).

## Data Availability

The data presented are properly cited and can be obtained from already published original research articles, which are available on electronic databases.

## Abbreviations

ARDS: Acute respiratory distress syndrome
CI: Confidence interval
HLH: Hemophagocytic lymphohistiocytosis
ICU: Intensive care unit
MERS-CoV: Middle east respiratory syndrome coronavirus
NOS: Newcastle–Ottawa scale
PRISMA: Preferred reporting items for systematic reviews and meta-analyses
RCT: Randomized controlled trial
RR: Risk ratio
SARS-CoV-2: Severe acute respiratory syndrome coronavirus 2
UK: United Kingdom
WHO: World Health Organization.
